# The Social Learning-Generosity (SL-Gen) Task: Redesigning the multi-round trust game paradigm to examine social learning and generosity sensitivity in young people

**DOI:** 10.3758/s13428-026-03100-2

**Published:** 2026-07-24

**Authors:** Brennan Delattre, Alister R. Dale-Evans, Amy L. Gillespie, Juliet D. Griffin, Erdem Pulcu, Susannah E. Murphy, Catherine J. Harmer

**Affiliations:** 1 Department of Psychiatry, University of Oxford, Warneford Hospital, Warneford Lane, Headington, Oxford, Oxfordshire OX3 7JX UK; 2 Oxford Health NHS Foundation Trust, Oxford, UK; 3 Department of Computer Science, University of Oxford, Oxford, UK

**Keywords:** Social learning, Trust game, Trust, Unfairness, Generosity, Young people, Task development

## Abstract

Social learning is important for societal functioning, and adolescence is a critical period for the development and maintenance of social behaviors. Specifically, the ability to establish and maintain beneficial social interactions while avoiding or withdrawing from detrimental or harmful ones is crucial for healthy interpersonal functioning. Beyond self-report measures, the multi-round trust game, a commonly used behavioral economics paradigm, can help elucidate differences in social decision-making between healthy and clinical populations. To more specifically probe the role of social learning and generosity sensitivity during social exchange in young people, we have designed a new task: the Social Learning-Generosity (SL-Gen) Task. Using versions of the multi-round trust game as a basis for the design, we have incorporated more environmentally realistic task components, including controlled gain, loss, and variable gain-loss generosity conditions. In a single-session online study, the present research investigated task behavior and task acceptability in individuals aged 18–24 from a United Kingdom census-based sample, as well as exploring task differences by self-reported trust and gender. While this task is sensitive to differences in self-reported trust and gender and behaves similarly to existing versions of the trust game with respect to these effects, it has added utility within mental health research and treatment contexts for characterizing social decision-making behavior in more detail using set generosity conditions.

**Supplementary Information:**

The online version contains supplementary material available at https://doi.org/10.3758/s13428-026-03100-2.

## Introduction

Decisions to trust or not trust others serve important functions at the interpersonal, community, and societal levels (Alós-Ferrer & Farolfi, 2019). Individuals’ ability to gauge others’ trustworthiness and analyze social signals from those around them, defined as components of *social learning,* fundamentally influences the quality of their interpersonal relationships (Fermin et al., 2022; Cook, 2001; Yamagishi, 2011). As adolescence and young adulthood are crucial periods of social development, accurately characterizing trust behavior during this period becomes critical to developing a baseline for comparison when examining social learning and trust decisions in clinical populations. Specifically, understanding trust-based decision-making in young people can help elucidate mechanisms of social function or dysfunction in situations and populations where social learning behavior is affected. The present research aimed to develop a task to better understand how young people learn and respond to different generosity behaviors in a social context.

### The trust game

**Fig. 1:**
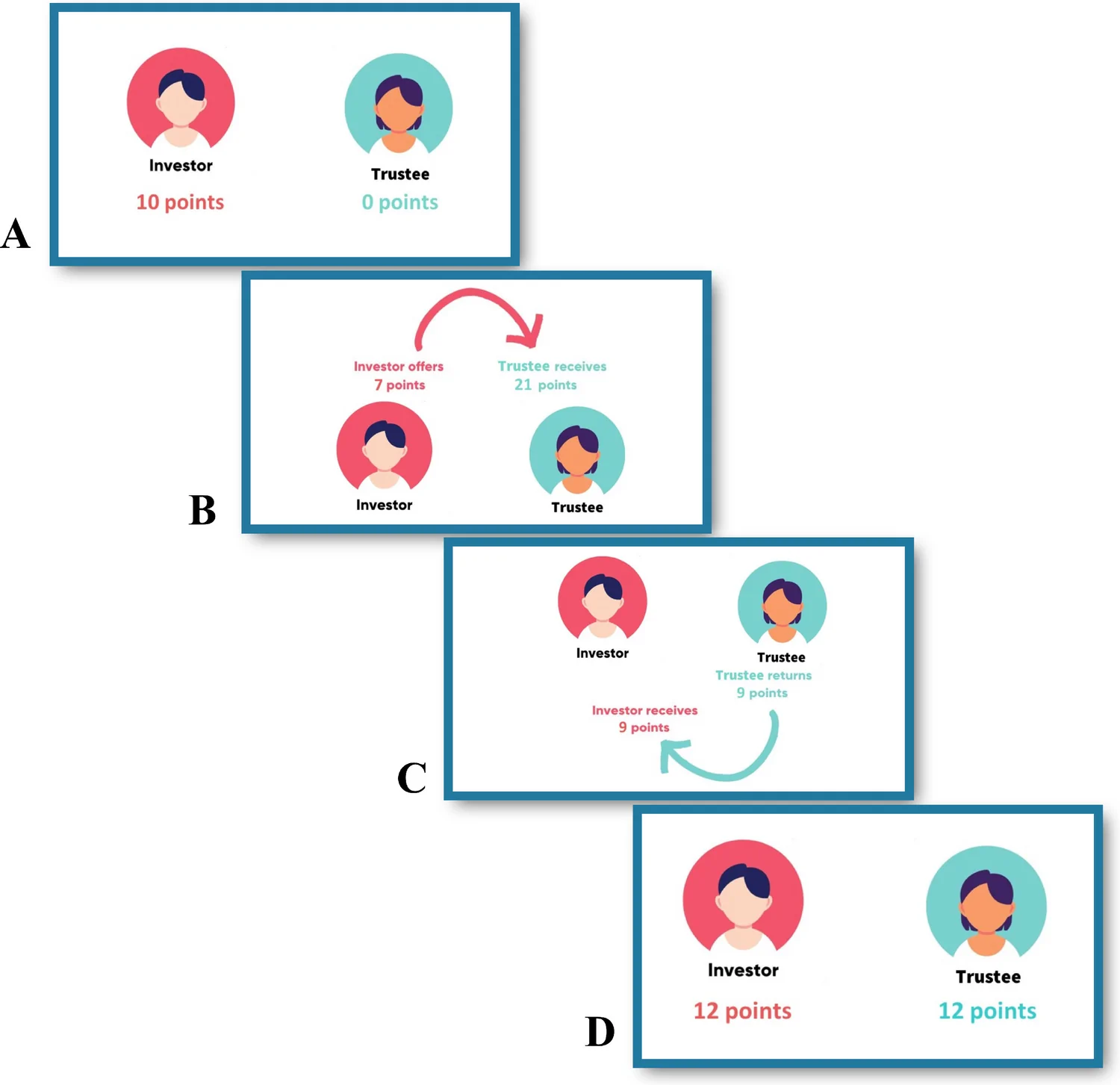
Classic trust game instructions. **A** There are two roles in the trust game: the investor and the trustee. Participants in this study all played the role of the investor. They played the game with twenty different trustees and ten rounds of the game with each trustee. At the beginning of each round of the game, the investor is given ten points to invest as they see fit; the trustee starts with zero points. **B** The investor then chooses how many of their ten points they would like to invest in the trustee. The invested points are then multiplied by three. In this example, if the investor sends seven points to the trustee, the trustee receives 21 points (7 x 3).** C** The trustee then decides how many (if any) of their points to return to the investor. The trustee keeps the points they do not return. **D** In this example, both players end up with more points than they began with. (Player 1: (10 original points – 7 invested) + 9 return = 12 total points, and Player 2: (0 initial points + (7 invested x 3)) – 9 returned = 12 total points.) Players can gain or lose similar or very different numbers of points depending on how much the investor chooses to invest and how much the trustee chooses to return. The aim of the game is to earn as many points as possible, and so participants, in the role of the investor, must therefore evaluate whether the trustee will return a fair proportion of the points they are given, and decide how much to invest accordingly

The trust game, a classic behavioral economics paradigm, has been used as a non-self-report measure of trust decisions in psychological and neuroscientific contexts for more than a decade (King-Casas & Chiu, 2012). In this two-player game, an individual assigned to the role of “investor” chooses to send any number of points between 0 and 10 to an individual in the role of the “trustee,” who receives triple the amount of points sent by the investor, and then decides how many points, if any, to return to the investor (Schreuders et al., 2024; Fig. 1)*.* The trust game can be played as a “single-shot” interaction, or it can be played iteratively, which is to say, with multiple rounds of points potentially exchanged between the two players. Researchers have assigned study volunteers to play the role of the investor in various versions of the trust game to date (for a selection of these, see Supplementary Table S1). Despite the difficulties and limitations of the trust game and other classic social decision-making paradigms (see Alós-Ferrer & Farolfi (2019) for review), task-based measures provide an elegant solution to probing factors of interest beyond direct self-report, and each existing version of the trust game has slightly different utility for examining individual and social factors in healthy and clinical populations.

### The trust game and demographic factors

Different aspects of identity, both internal and intergroup (e.g., ingroup versus outgroup factors) can influence social learning and trust game behavior (Vermue et al., 2018). For instance, a recent meta-analysis of gender differences in trust game behavior found that men invest more money than do women in contexts where their points will be multiplied, creating an efficiency gain (van den Acker et al., 2020). Men have also been suggested to take more social risks than women across cultures, contexts, and ages, which has been suggested to reflect cultural and evolutionary tendencies for men to take more risks and behave more competitively and independently, whereas women are typically socialized to behave in a more nurturing and communal way (van den Acker et al., 2020). These tendencies could translate to investment behavior in social exchange task contexts. Additionally, a recent meta-analysis of UK-based social survey participant data suggested that trust game behavior has been found to predict generalized self-reported trust significantly and positively (Banerjee et al., 2021).

### The trust game and clinical populations

Existing literature suggests that individuals with mental health difficulties, including those with depression, make different decisions on the trust game and other cooperative game-based tasks when compared to the decisions of control populations. However, the directionality and details of these differences are not yet entirely clear. Specifically, some studies have indicated that individuals with depression exhibit increased cooperation on tasks of this nature, while others suggest individuals with depression exhibit disrupted cooperation, such as the inability to sustain cooperation, instead (Ong et al., 2017; Mellick, 2014; Mellick et al.,^2019^; McClure et al., 2007; Clark et al., 2013). It is also not clear, from the existing social exchange literature, whether participants with depression invest more than control populations to try to incentivize others to be more generous with them, or if, when confronted with unfair exchanges or uncooperative partners, depressed individuals do not realize they should cease or limit investments with those whom evidence suggests will offer poor returns on investments. Due to the diversity of the ways the trust game has been implemented and the broad context of the task, it is also possible that the different task implementations contributed to the different directionalities and variety of behavioral results.

In light of the disruptions and impairments in social functioning that are present in depression and other mental health disorders (King-Casas & Chiu, 2012), investigating social learning, decision-making, and interpersonal generosity sensitivity in individuals with depression could help elucidate potential mechanisms underlying these difficulties. Specifically, the present work seeks to characterize two related processes: social learning and generosity sensitivity. Social learning refers to the process by which individuals update their expectations and adapt their behavior over repeated social interactions based on feedback from others’ actions. In a multi-round trust game context, this is reflected in how participants modify their investment behavior across rounds as they learn whether specific partners tend to behave fairly or unfairly. By contrast, generosity sensitivity refers to the degree to which individuals perceive and respond to the magnitude of others’ fairness or unfairness—that is, how strongly they evaluate, differentiate, and react to being treated generously versus ungenerously.

Although these mechanisms are closely intertwined in social exchange, they are not identical: generosity sensitivity captures evaluative and perceptual responses to partner behavior, whereas social learning captures behavioral adaptation over time. Importantly, many observable behaviors in social exchange tasks likely reflect the interaction of both processes rather than either one in isolation.

To better holistically examine social learning and generosity sensitivity, potentially critical mechanisms of social exchange differences in populations with depression, we sought to draw upon existing multi-round trust game paradigms to develop a more environmentally realistic multi-round social learning task using more ecologically valid controlled generosity conditions, as well as more realistic task stimuli. We then tested the task for self-reported trust and gender effects and investigated its acceptability using quantitative and free-response participant feedback items. To maintain clarity of scope, the current report details the development, structure, and behavioral validation of the Social Learning-Generosity (SL-Gen) task. A subsequent companion paper analyses associations between task performance and mental-health variables, including depression.

This study was guided by several theoretical expectations. First, if the SL-Gen task captures behavior relevant to generosity sensitivity, participants should differentiate between trustee generosity conditions, investing more with trustees who return more points and less with trustees who return fewer points, and updating generosity ratings in the expected direction after repeated interactions. Second, if the task captures feedback-driven social learning, investment behavior should change over rounds as participants accumulate information about each trustee, with decreasing investment in loss conditions and more stable or increasing investment in gain conditions. Third, given prior evidence linking trust-game behavior with self-reported trust and gender, we expected SL-Gen task behavior to show sensitivity to these individual-difference variables. Finally, because the present manuscript focuses on task development and validation, we also examined task believability, engagement, and reported strategy use to assess whether the paradigm was acceptable and credible for future use in experimental and clinical research. These expectations guided the design and analysis of the present study, which remained exploratory in that it was not preregistered and was intended to establish baseline behavioral patterns for future work.

## Methods

### The task redesign

**Fig. 2:**
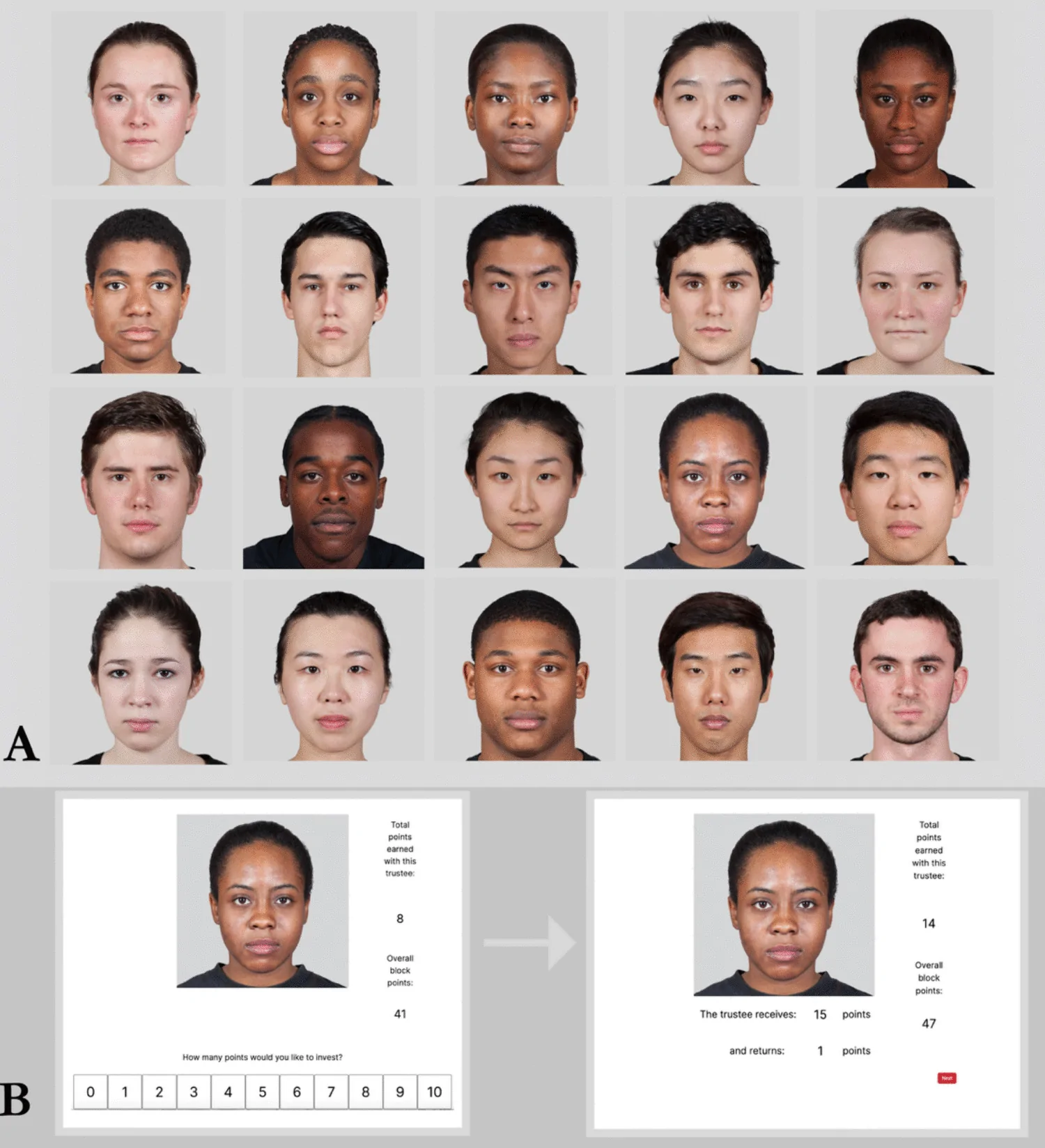
The Social Learning-Generosity (SL-Gen) task. **A** Twenty images from the MR2 Face Bank were used to make this version of the trust game’s stimuli more ecologically relevant to participants in terms of age, ethnicity, and photorealism. **B** Example task screens. The *left panel* shows the investment-choice phase, in which the participant (investor) decides how many of their ten points to invest with the trustee. The text on the right side of this first panel displays cumulative point totals, where “Total earned with this trustee”—in this case, eight points—is the total earned from previous rounds with this specific trustee, and “Overall block points” is the points the participants have earned over all rounds they have played during the block (across trustees). The participant is asked, “How many points would you like to invest?” using the 0–10 scale on the bottom of the screen. After the participant makes a selection, the right panel presents the outcome feedback screen, which displays how many points the trustee received—in this case, the participant invested five points, and the trustee received 15—and how many the trustee returned (in this case, one point). Participants therefore learn about each trustee’s behavior gradually through repeated feedback across rounds

To characterize social learning behavior and generosity sensitivity in young people with and without depression, we redesigned the multi-round trust game paradigm with the aim of increasing ecological validity, resulting in the Social Learning-Generosity (SL-Gen) task (Fig. 2). This task uses both more diverse (multiracial) and realistic (real people) face stimuli from the MR2 face bank (Strohminger et al., 2016) to represent other players in the game.

**Fig. 3:**
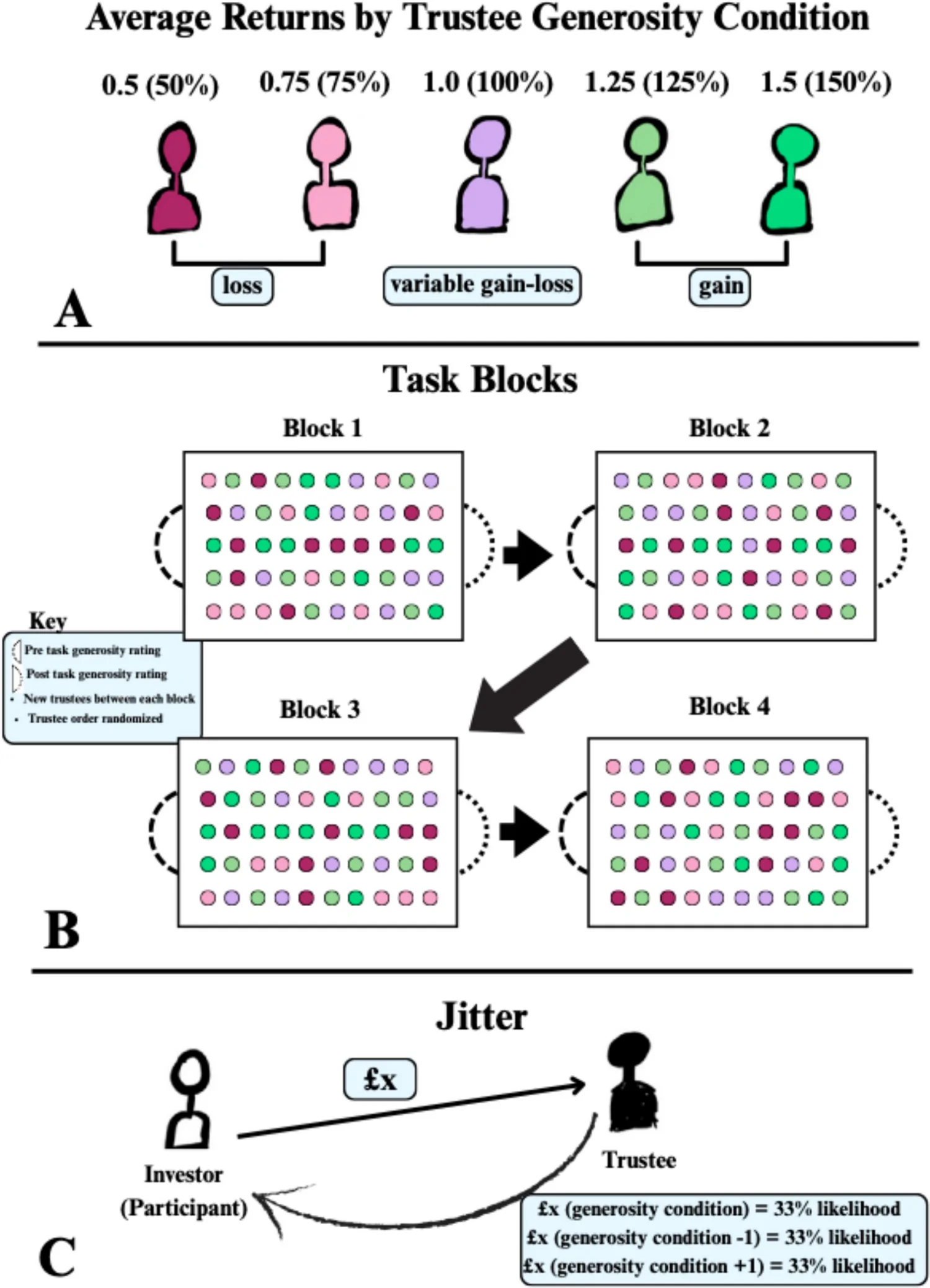
Generosity conditions and blocking in the SL-Gen task.** A** This task employs five generosity conditions: the trustee returns approximately 1) 50%, 2) 75%, 3) 100%, 4) 125%, or 5) 150% of the investor’s investment. **B** Trustees were divided into four blocks**,** each containing one trustee representing each generosity condition (five trustees per block). Participants complete ten rounds with each trustee, for a total of 200 rounds across the full task. Within blocks, trustees are presented in a randomized, interleaved order, such that participants cannot predict when they will see a particular trustee. Over the course of one game, an investor will play with four trustees of each generosity condition. To reduce the potential for bias, each time the task is generated, trustee face images are assigned randomly to blocks and generosity conditions. **C** To make trustee behavior more realistic, the task includes jitter, where each trustee return is rounded to the nearest whole number and there is 33% probability that the trustee will return exactly that number of points, that number plus one point, or that number minus a point (e.g., if an investor invests five points with a 100% generosity trustee, the trustee has an equal chance of returning four, five, or six points).

It also uses pre-programmed trustee generosity conditions (divided into gain, variable gain/loss, and loss versions) that more realistically model the gamut of behaviors young people may encounter and need to respond to (e.g., interactions with individuals who behave very generously, a little generously, variably, a little ungenerously, or very ungenerously; Fig. 3).

Specifically, we elected to use asynchronous, pre-programmed trustees, and in our paradigm, all research participants play the role of the investor. As we were predominantly interested in peer relationships and interactions in young people aged 18–24, faces of young people in a similar age category (ages 18–25) were selected from the MR2 face set to represent trustees. This set comprises standardized, validated photographic stimuli of individuals with European, African, or East Asian ancestry displaying neutral expressions (for baseline trustworthiness ratings of faces, please see Supplementary Figures S1 and S2). Participants in this task are instructed that they will play the game with randomly selected former participants from a previous study who played in the role of the trustee; while the trustee decisions are not live, they are the responses these trustees gave to former participants who invested the amount that the participant chooses to invest. This cover story states that, while the trustees were real previous participants, they provided their answers asynchronously.

In reality, we created five different generosity conditions (two “gain” conditions, one “variable gain-loss” condition, and two “loss” conditions): 1) 1.5: in which the most generous trustees returned on average 150% of the participant’s investment; 2) 1.25: in which the somewhat generous trustees returned on average 125% of the participant’s investment; 3) 1.0: in which the variable gain-loss trustees returned exactly the participant’s investment a third of the time, a point more a third of the time, and a point less a third of the time; 4) 0.75, in which the somewhat ungenerous trustees returned on average 75% of the participant’s investment; and 5) 0.5, in which the very ungenerous trustees returned on average 50% of the participant’s investment (Fig. 3A). Each condition is allocated four trustee faces, and participants play ten rounds with each trustee in a randomized, interleaved order, such that participants play 40 rounds with each generosity condition, or 200 rounds in total (Fig. 3B). Each investment decision is followed by feedback indicating the trustee’s return for that round. The block design was introduced to make the learning demands feasible: interacting with 20 trustees simultaneously may make it difficult for participants to remember each trustee’s behavior. Instead, by limiting each block to five trustees, participants learn about a smaller, manageable subset at a time while still encountering all generosity conditions within each block. Interleaving trustee presentation order within the blocks was done to reduce order effects and encourage learning based on observed trustee behavior rather than presentation sequence. This structure allowed participants to remember and update their impressions of each trustee’s behavior over repeated interactions, supporting meaningful social learning within cognitively realistic constraints. On each trial, our algorithm calculated the trustee’s base return as the participant’s current investment multiplied by the trustee’s assigned generosity parameter, after which jitter was applied to add realism and unpredictability. As participants are told that they are playing asynchronously with real people who previously participated in studies in the lab, we utilized jitter—specifically, uniformly distributed discrete noise—to ensure that trustees behave more realistically by not returning precisely the same point value each time the same amount is invested. Jitter was achieved by randomizing trustee responses in such a way that there is an equal probability that the trustee returns exactly the value of their generosity condition, or generosity condition response plus/minus one point (Fig. 3C). In selecting the generosity conditions for this task, it was crucial to include loss conditions, such that in some conditions participants receive fewer points back than they invest, so as to be able to examine behavior in light of unfair and/or disadvantageous exchange conditions.

While several previous versions of the multi-round trust game have examined related processes—including facial trustworthiness perception (Chang et al., 2010), binary trust decisions (Van Den Bos et al., 2011) and facial trustworthiness (Ewing et al., 2015) in developmental samples, and ingroup versus outgroup decision-making using symbolic partners (Vermue et al., 2018, 2019)—the Social Learning-Generosity (SL-Gen) task builds on and extends this work by combining the controlled structure of a pre-programmed multi-round trust game with enhanced ecological validity. In contrast to earlier paradigms, the SL-Gen uses realistic, demographically diverse facial stimuli rather than symbolic or limited face sets, randomizes faces to generosity conditions to avoid confounding facial appearance with behavior, and employs a continuous investment scale that allows fine-grained analysis of learning over time. This design is intended to support partner-specific expectations and investment adaptation based on consistent feedback, which are relevant to studying social learning and generosity sensitivity in both healthy and clinical populations. As many observable social exchange behaviors reflect the interaction of social learning and generosity sensitivity rather than either mechanism in isolation, the SL-Gen task was designed to provide a set of complementary outcome measures that together characterize how individuals perceive, evaluate, and adapt to others’ generosity across repeated interactions, rather than to isolate any one mechanism conclusively within a single analysis.

Although previous versions of the multi-round trust game have also used asynchronous, pre-programmed trustees, the SL-Gen task is distinct in its focus on learning from feedback about others’ fairness rather than on real-time reciprocal exchange. Participants are told that the trustees represent previous participants whose responses were recorded in earlier studies, and that they are now observing those responses. Because trustee returns are not dynamically contingent on participants’ decisions, the task does not measure strategic reciprocity or trustworthiness in the behavioral-economics sense. Instead, it probes social learning and generosity sensitivity—that is, how individuals evaluate, learn about, and adapt to partners who behave more or less generously over repeated interactions. We therefore refer to the trustee return parameter as *generosity* rather than *reciprocity*, as it reflects an inherent behavioral tendency of each trustee (returning approximately 50–150% of the investment) rather than a negotiated or strategic response. This design allows the SL-Gen task to probe learning processes related to fairness and generosity while maintaining the classic trust-game framework of sequential investment and feedback.

### Participant sample

We conducted a single-session online research study with young people aged 18–24 to determine how they made decisions in the SL-Gen task for validation and task acceptability. Individuals aged 18–24 living in the United Kingdom were recruited using Prolific’s (www.prolific.com) standard United Kingdom census-representative sample option, which draws upon 2024 census data for England & Wales, Northern Ireland and Scotland to include participants by ethnicity (using the five categories represented by the UK Office of National Statistics: White, Mixed, Asian, Black, and Other), age (18–24) and sex (stratified into male and female). Inclusion criteria included having access to a computer with internet access, not having already participated in the study, and being able to read and write English proficiently. As this research sought to establish a baseline of task behavior, individuals who self-identified as having a current or recent diagnosis of, or having received treatment for, a substance use disorder, bipolar disorder, a psychotic disorder, an eating disorder, or a personality disorder were excluded.

A total of 572 individuals participated in this study; of those, ten were excluded for finishing the SL-Gen task faster than was possible without clicking random buttons and, when asked to provide information on their investment strategy as a sensibility check, were unable or chose not to respond. A further nine individuals’ data were excluded due to server connectivity issues that resulted in them playing more or less than 40 rounds per trustee generosity condition. Ultimately, 553 participants (*M* = 21.9 years, *SD* = 1.67) completed the social learning task and were included in the analysis. When asked to report their gender, 267 participants selected “Female”, 274 selected “Male”, and 12 selected “Nonbinary or other (please specify)”^1^. For further demographic information, including ethnicity, education, and household income, please see Supplementary Table S2.

### Measures

#### Task measures

The primary outcome measures of SL-Gen task performance were divided into two categories and quantified as follows:

1. How do individuals behave in the game?
  a Total points earned: the absolute value of the points participants gained or lost in the game;
  b Initial investments: the average of the first investments made with each of the five trustees encountered in the first block. Because each block included one trustee from each generosity condition, these five trials were selected to capture participants’ first encounters with novel trustees before receiving direct multi-round feedback about trustees’ return behavior, providing an index of early investment tendencies before meaningful task-specific learning had occurred;
  c Investments by trustee generosity condition: the total points invested over the game with each type of trustee (loss (0.5 and 0.75 conditions), variable gain/loss (1.0 condition), and gain (1.25 and 1.5 conditions));
  d Change in trustee generosity ratings: the absolute value of the change in anticipated trustee generosity rating pre-exchange to actual trustee generosity rating post-exchange.
2. How does behavior change over the course of the game?
  e Change in investment behavior over rounds***:*** the slope of investment behavior by trustee generosity condition over the course of the game.
  f Investment plateaus: the average point value of the last three investments by trustee generosity condition. This window was selected as a pragmatic index of late-stage investment behavior after accumulated feedback, while preserving the earlier trials as the primary period during which learning and behavioral adaptation could occur. Averaging across the final three investments yielded a more stable estimate of end-stage behavior than a single final trial, while preserving earlier rounds for exploratory learning.
  g Desisting from investing: the count of zero investments (e.g., when participants choose not to invest any points) by trustee generosity condition and round number.

Measures were selected to index distinct phases of behavior within the learning process, including early investment tendencies, behavioral adaptation over time, late-stage stabilization, and withdrawal from disadvantageous exchanges. In terms of the link between these outcome measures, social learning, and generosity sensitivity, these measures provide complementary lenses for both processes rather than attempting to separate them in a one-to-one manner. Some outcome measures are more proximal to social learning, whereas others are more proximal to generosity sensitivity, but all are likely influenced by both. Specifically, initial investments index early decision-making and baseline risk-taking before meaningful feedback has been accumulated. Investments in the trustee generosity condition reflect both social learning and generosity sensitivity, as the number of overall points participants invest in each condition over the course of the game is likely to be informed by both factors. Changes in investment behavior across rounds (investment slopes) capture how participants adapt their behavior in response to experienced trustee generosity and are more directly related to social learning. The investment plateau reflects late-stage stabilization of behavior once expectations about trustee behavior have formed and is also more directly related to social learning, addressing where behavior stabilizes once learning has occurred. Desisting from investing captures whether and when participants learn to disengage entirely from exchanges with disadvantageous trustees, and reflects both social learning and generosity sensitivity, as choosing to cease investing with a trustee could be primarily a product of sensitivity to ungenerous behavior or primarily a product of learning that the trustee is not a good investment. Changes in trustee generosity ratings index evaluative updating of perceived partner generosity based on experienced behavior; this variable is most directly related to generosity sensitivity, as it captures evaluative updating of others’ perceived behavior. However, none of these outcomes is a pure measure of either construct, and the present single-session cross-sectional study is intended to characterize how these social learning and generosity sensitivity co-occur and jointly shape investment behavior across repeated interactions. Together, these measures provide a multi-dimensional characterization of how participants perceive, evaluate, and adapt to others’ generosity across repeated social interactions, rather than relying on any single outcome in isolation.

#### Demographic and questionnaire measures

Participants provided demographic information (age, gender, ethnicity, education level, and household income) and responded to the following self-report trust questions used in previous research investigating the link between trust game behavior and self-report trust:

1. the trust item from the General Social Survey (Banerjee et al., 2021): “Generally speaking, would you say that most people can be trusted or that you need to be very careful in dealing with people” (with answer measured on an 11-point Likert scale where 0 represents “You need to be very careful in dealing with people” and 10 represents “Most people can be trusted.”);
2. the OECD Generalized Trust Question (Rosenberg et al., 1956): “In general, how much do you trust most people?” with answer measured on an 11-point Likert scale from “Not at all” to “Very much”; and
3. the hypothetical lost wallet question: “If you lost a wallet or purse that contained items of great value to you, and it was found by a stranger, do you think it would be returned with its contents or not?” (binary choice between “I think it *would* be returned” or “I think it would *not* be returned).

Participants also completed a broader set of mood and mental-health questionnaires, the results of which are reported separately in a companion manuscript focusing on clinical outcomes.

#### Task acceptability and feedback measures

To assess task acceptability and participant perceptions, feedback items were administered immediately after the SL-Gen task. Participants rated the *believability* of the cover story (“While playing the game, did you believe that you were genuinely playing with past research participants, or something else?”) on a slider from −10 (“I definitely did not believe I was playing with past research participants”) to +10 (“I definitely believed I was playing with past research participants”), with an optional free-text response (“If you believed something else, what did you believe?”). Participants were also asked whether any trustee faces were *distinctive or memorable* (“Were there any faces you were more drawn to than others? Any that were memorable for a specific reason?”; open-ended response). Task *engagement* was rated on two continuous scales: one assessing how engaging the task was (0 = “Extremely unengaging” to 100 = “Extremely engaging”) and another assessing whether participants were using a strategy versus responding at random (0 = “Just pushing buttons to get the task to end as quickly as possible” to 100 = “Genuinely using a strategy to win as many points as possible”). Finally, participants were invited to indicate what they thought the *purpose of the study* was (“What do you think we were studying in this research?”; open-ended response). Quantitative feedback ratings were analyzed descriptively, and qualitative responses were thematically coded.

### Procedure

This single-session study was approved by Oxford University Central University Research Ethics Committee (CUREC; Reference: R89065/RE001) and was conducted online in March 2024. Questionnaires and tasks were hosted on Gorilla (www.gorilla.sc; Anwyl-Irvine et al., 2020) and participants were recruited via Prolific. Participants were provided with a brief description of the study, as well as estimated participation time and compensation rate. They were told they would be asked to play an investment game with other previous research participants to try to gain as many points as possible, as well as to fill out questionnaires about how they feel and other demographic information. They then confirmed that they met the inclusion criteria and provided consent.

Next, participants completed baseline measures, including demographic information, questionnaires, and self-report trust questions. Participants also completed a battery of self-report questionnaires assessing mood, trust, and related mental-health variables; however, analyses linking these clinical measures to SL-Gen task performance are reported separately, whereas the present manuscript focuses on task development, validation, and behavioral characteristics of the sample.

Participants received task instructions for the SL-Gen task, in which they were told that their primary goal was to maximize their points; that they would be playing the task with some of the lab’s past participants; and that just like any people, some of these past participants were more generous than others. Participants next completed a comprehension quiz, followed by the SL-Gen task, which lasted approximately 15 min. After the task, participants again completed questionnaires and provided feedback on the task before being directed back to Prolific at the end of the study. Participants received £5.25 for completing the study, which took approximately 27 min to complete. Given the exploratory nature of this study and the number of variables analyzed, we opted for a large sample size—considerably larger than most experimental medicine studies where tasks of this nature are used. We sought to have sufficient statistical power to provide preliminary evidence for the validation of this task to be used in clinical and other experimental contexts. A post hoc sensitivity analysis revealed that the sample size (*n* = 553) would provide 80% power at an alpha level of.05 to detect a minimum effect size of Cohen’s *f* = 0.13 in an ANOVA contrasting three groups, and a minimum effect size of *r* =.12 for a two-tailed Pearson correlation.

### Statistical analysis approach

Analyses were organized around the SL-Gen task outcomes described above. Full details of the dataset, model type, predictors, interactions, and random-effects structure for each analysis are provided in Supplementary Table S3. Analyses were conducted in R (version 4.1.3), and all data and analysis code are available in the open repository listed in the Code availability section.

Specifically, participant-level outcomes, including net points earned and initial investments, were analyzed using ANOVA or linear regression models to test associations with gender and self-reported trust. Total investments were examined separately by trustee generosity condition to test whether participants invested differently with loss, variable gain/loss, and gain trustees, and whether these condition-specific investment totals differed by gender or self-reported trust. The trustee generosity condition was treated as categorical, as the five levels (0.5, 0.75, 1.0, 1.25, 1.5) were designed to represent distinct social exchange contexts rather than a single continuous predictor. Self-reported trust was analyzed continuously in regression models and grouped into quartiles for visualization and selected group comparisons.

Pre- to post-task generosity ratings were compared within each trustee generosity condition using paired-samples t-tests to assess whether participants updated their ratings in the expected direction after interacting with each trustee type. Follow-up ANOVA models were used to examine whether rating change differed by gender or self-reported trust. To learn over time, investment slopes were calculated for each participant to summarize changes in investment behavior across rounds, and the slopes were then analyzed using linear mixed-effects models with participant random intercepts to test whether learning trajectories differed by trustee generosity condition, gender, or self-reported trust. Interactions were included in these slope models when they tested whether adaptation to trustee generosity differed by gender or self-reported trust.

Investment plateaus were analyzed separately by trustee generosity condition to examine late-stage investment behavior and group differences by gender or self-reported trust. Desisting from investing was analyzed descriptively by calculating the percentage of participants continuing to make non-zero investments across rounds, trustee generosity conditions, gender, and self-report trust groups. This analysis was included to characterize withdrawal from disadvantageous exchanges rather than as a formal inferential test.

## Results

Results are organized to reflect different stages of behavior in the SL-Gen task, beginning with overall measures (points earned) and early investment tendencies (initial investment decisions), followed by behavioral differentiation across generosity conditions (investments by trustee generosity condition and change in trustee generosity ratings), and culminating in analyses of learning dynamics over time, including adaptation (investment slopes), stabilization (investment plateaus), and disengagement (desisting from investing). Across these outcomes, patterns were broadly consistent with behavior reflecting both social learning and generosity sensitivity.

### Self-report trust

Participants responded to three self-report trust items prior to the social learning task: the trustworthy or careful question from the General Social Survey; the OECD Generalized Trust Question; and the hypothetical lost wallet question. While participant responses to the trustworthy or careful question (*M* = 4.65, *SD* = 2.18, median = 5, range = 0–10) and general trust question (*M* = 5.03, *SD* = 2.05, median = 5, range = 0–10) were fairly evenly distributed, 65.3% of participants believed that a lost wallet would not be returned to them, versus only 34.7% that believed it would be returned.

To assess the relationship between the Trustworthy or Careful Question and the General Trust Question, a Spearman’s rank-order correlation was conducted. The analysis revealed a strong positive correlation between the two variables, *r*_*s*_(553) =.740, *p* <.001. This suggested that participants who reported higher levels of trust in most people (versus needing to be very careful in dealing with people) also tended to report that most people can be trusted in general.

To further evaluate the internal consistency between these two variables, Cronbach’s alpha was calculated, yielding a coefficient of *α* =.851. This high level of internal consistency indicated that the Trustworthy or Careful Question and the General Trust Question were likely measuring the same underlying construct. Given the strong correlation and high internal consistency, the two variables were combined into a single composite variable by taking the mean score of the two variables per participant; this new variable was termed Self-Report Trust. Of the 553 participants, 110 participants fell into the highest self-report trust quartile; 279 participants were in the two middle quartiles; and 164 participants fell into the lowest quartile.

### Social Learning-Generosity (SL-Gen) task outcomes

The first analyses examined overall task performance and early investment behavior before substantial trustee-specific learning had occurred.

#### Points earned

Participants were instructed that the goal of the game was to earn as many points as possible. To examine which participants played the game most optimally based on these instructions, participants’ net total point outcome was calculated as their final point score minus 2000 points—the number of points participants would earn in the game if they made no investments (10 points per round and 200 rounds of the game). Overall, participants earned 96.7 points on average (*SD* = 74.3; median = 105). Two one-way ANOVAs were conducted to examine whether self-report trust and gender significantly predicted the net points participants earned in this task. While self-report trust did not significantly predict the net points participants earned in the task (*F*(1, 551) = 0.245, *p* = 0.621), there was a significant main effect of gender, *F(*2, 550) = 11.53, *p* <.001, with a small to medium effect size (η^2^ =.04, 95% CI [.02, 1.00]), indicating that approximately 4% of the variance in net points earned can be explained by gender. Post hoc Tukey’s HSD comparisons revealed that male participants earned significantly more points in this task (*M* = 111.75, *SD* = 72.37) than did female participants (*M* = 81.88, *SD* = 73.59, *p* <.001, mean difference = 29.87, 95% CI [15.14, 44.60]). However, there was no significant difference between nonbinary or other and female participants (*p* =.991) or male participants (*p* =.416).

#### Initial investment decisions

To examine the risk-seeking versus risk-averse behavior of individuals in this social learning context, and to determine whether gender or self-report trust predicted initial investment decisions of participants before they had meaningfully interacted with the trustees in the social learning task, participants’ initial investments were averaged from the first five investments they made in the first round with the first five trustees they encountered in the task. A one-way ANOVA revealed that gender predicted participants’ initial investments in the task; specifically, there was a significant main effect of gender, *F*(2, 550) = 6.36, *p* =.0019, with a small effect size (η^2^ =.02, 95% CI [.01, 1.00]), which suggested that about 2% of the variance in initial investments is explained by gender. Tukey’s HSD comparisons showed that male participants (*M* = 5.72, *SD* = 2.05) invested significantly more than female participants (*M* = 5.15, *SD* = 1.72) in the first five rounds (*p* =.0013, mean difference = 0.57, 95% CI [0.19, 0.95]), and that there was no significant difference between nonbinary or other participants and female participants (*p* =.548) or male participants (*p* =.9998). Another one-way ANOVA revealed that self-report trust predicted participants’ initial investments, (*F*(3, 551) = 24.46, *p* <.001). A linear regression of self-report trust controlling for gender to predict initial investments accounted for approximately 6.47% of the variance in initial points invested, and higher levels of self-report trust corresponded with higher initial investments (*B* = 0.197, *SE* = 0.040, *t*(549) = 4.97, *p* <.001).

Next, we examined whether participants differentiated between trustee generosity conditions in both investment behavior and post-task generosity ratings.

#### Investments by trustee generosity condition

To examine the gross variation in individuals’ investments between trustee generosity conditions, each participant’s total investment per trustee generosity condition (averaged across the four blocks) was calculated. As anticipated, trustee generosity condition predicted investment at each level (*F*(4,411109) = 10408, *p <.*001), and specifically, the mean investment per trustee generosity condition increased in correspondence with increased trustee generosity (0.5, 0.75, 1.0, 1.25, 1.5; Fig, 4a). A series of linear regressions was conducted to determine whether self-reported trust predicted participant investment across the different levels of the trustee generosity condition. Self-report trust did significantly predict mean investment in every trustee generosity condition (0.5: *F*(1,551) = 4.70, *p* =.031; 0.75: *F*(1,551) = 10.76, *p* =.001; 1.0: *F*(1,551) = 21.27, *p* <.001; 1.25: *F*(1,551) = 14.83, *p* <.001; 1.5: *F*(1,551) = 7.95, *p* =.005). In each case, more self-report trust was associated with higher point investments (Fig. 5a). ANOVAs were also conducted to determine whether gender predicted participants’ point investments for each trustee generosity condition (0.5, 0.75, 1.0, 1.25, 1.5). While there was no significant effect of gender on point investment in the loss conditions (0.5: *F*(2, 550) = 1.06, *p* = 0.35; 0.75:* F*(2, 550) = 0.51, *p =* 0.602), these analyses revealed a significant effect of gender on point investment in the variable gain-loss condition (1.0: *F*(2, 550) = 4.62, *p =* 0.01) and the gain conditions (1.25: *F*(2, 550) = 9.67, *p* <.001; 1.50: *F*(2, 550) = 12.19, *p* <.001; Fig. 6a); specifically, male participants invested more points in the variable gain-loss and gain conditions.

#### Change in generosity ratings

At the beginning of each block, participants rated how generous they thought each trustee would be based on just the image of the trustee; directly after completing ten rounds of play with all trustees in the block, participants rated how generous they thought each trustee actually was. These ratings were entered by typing a number between 1 and 100, where 1 indicated “Very ungenerous” and 100 indicated “Very generous.” The average change from pre-task generosity ratings to post-task generosity ratings per generosity condition per participant was calculated by subtracting the pre-task generosity rating from the post-task generosity rating per generosity condition per participant.

**Fig. 4:**
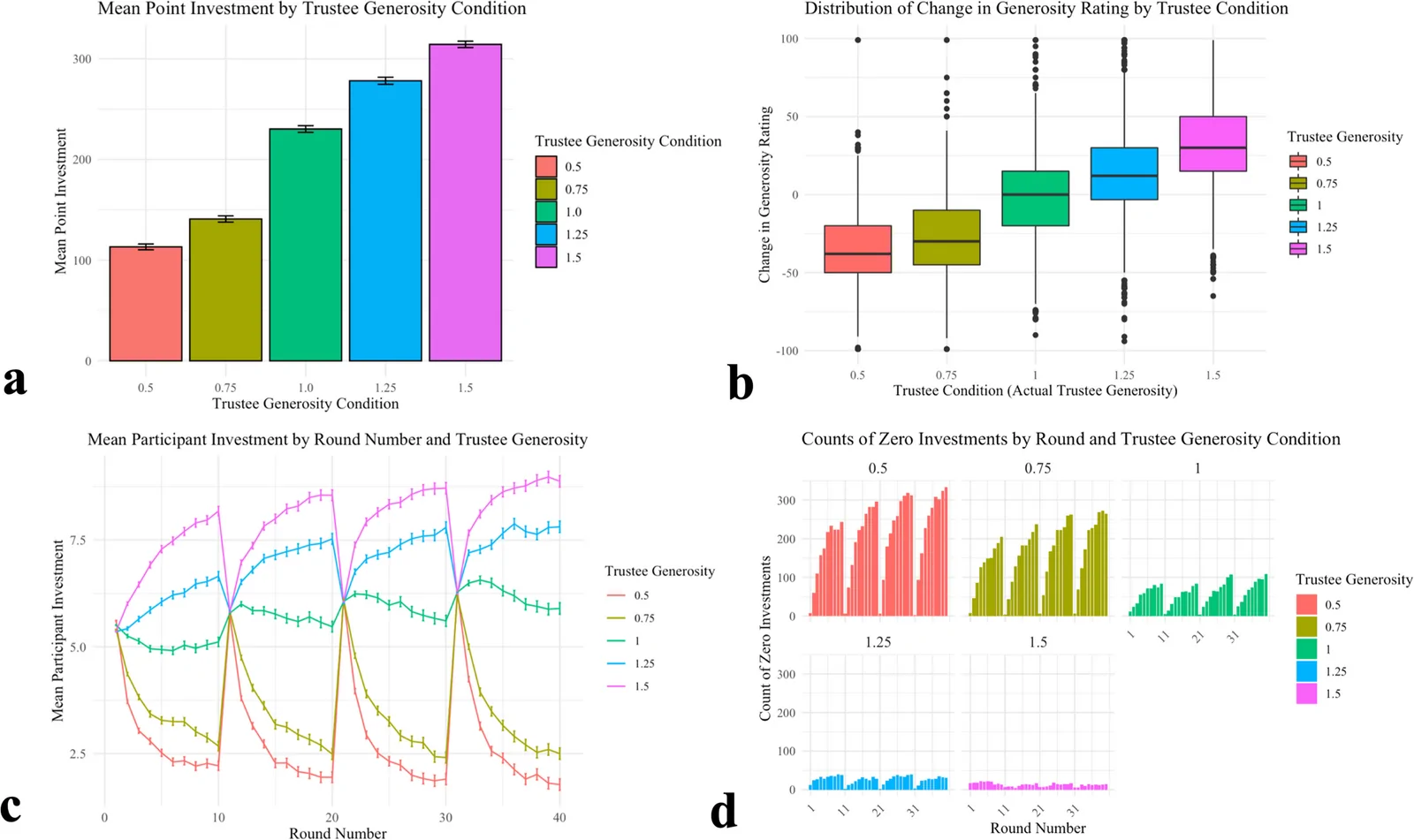
**a** Overall mean point investment by trustee generosity condition; **b** distribution of change of generosity rating by trustee generosity condition; **c** mean participant investment by round number and trustee generosity condition for all participants; **d** zero investments over rounds by trustee generosity condition

Paired-samples *t* tests were conducted to compare participants’ pre-game and post-game generosity ratings across the different levels of trustee generosity condition. In all trustee generosity conditions, there was a significant difference between pre- and post-task generosity ratings. Specifically, for the generosity conditions where trustees always returned fewer points than they were given by participants (namely 0.50 and 0.75), paired *t* tests revealed a significant decrease in generosity ratings in both the 0.50 (*t*(552) = – 49.5, *p* <.001) and 0.75 (*t*(552) = – 37.0, *p* <.001) conditions. In the conditions where trustees always returned more points than they were given by participants (1.25 and 1.50), there was a significant increase in generosity ratings in both the 1.25 (*t*(552) = 16.92, *p* <.001) and 1.50 (*t*(552) = 37.6, *p* <.001) conditions. In the variable gain-loss generosity condition (1.0, where trustees sometimes returned slightly more, exactly the same, or slightly less than the participant invested with them) there was a statistically significant decrease in generosity ratings from pre- to post-task (*t*(552) = – 3.60, *p* <.001); Fig. 4b.

**Fig. 5:**
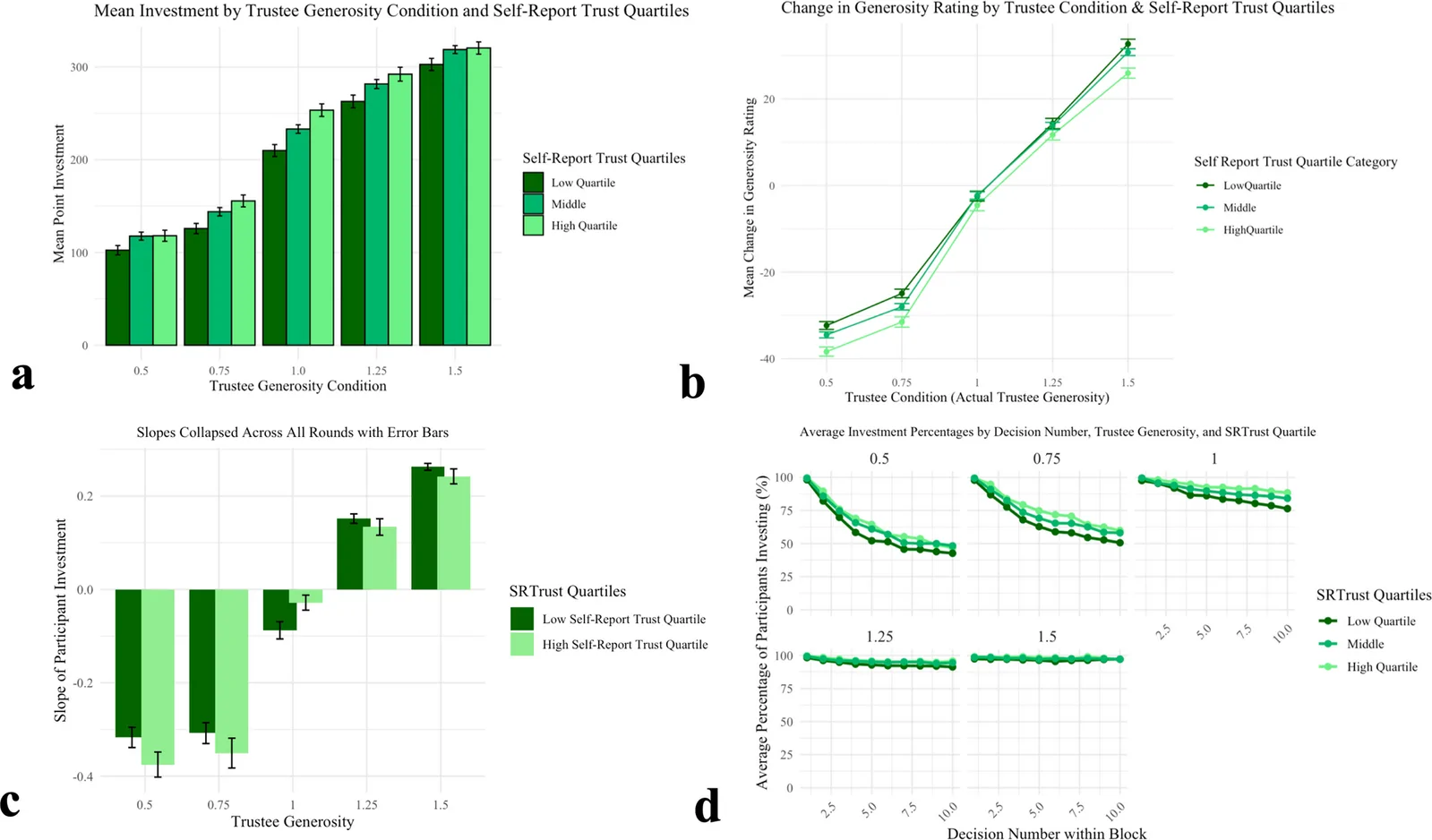
**a** Mean investment by trustee generosity condition and self-report trust quartiles. *Note:* while investments by trustee generosity condition are represented graphically by self-report trustee generosity quartiles, statistical tests examine self-report trust as a continuous variable; **b** Change in generosity rating by trustee condition & self-report trust quartiles; **c** Investment slopes aggregated across all rounds for trustee generosity condition by self-report trust quartiles; **d** Percentage of individuals still investing a non-zero number of points in the task by self-report trust quartiles

**Fig. 6:**
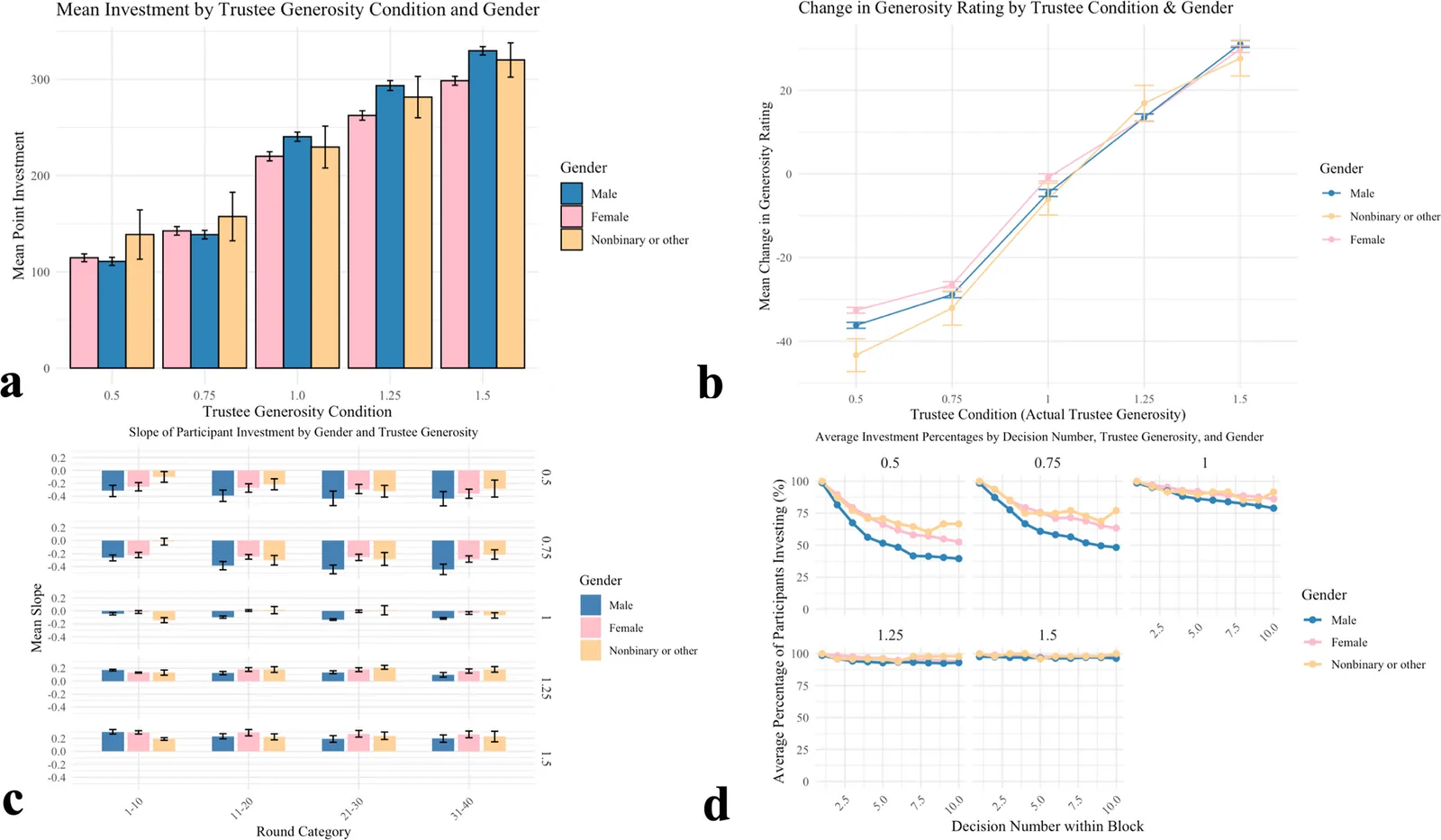
**a** Mean investment by trustee generosity condition and gender; **b** Change in generosity rating by trustee condition & gender; **c** Investment slopes by round ranges for trustee generosity condition by gender; **d** Percentage of individuals still investing a non-zero number of points in the task by gender

An ANOVA determined that self-report trust quartile significantly predicted change in generosity rating *(F*(2,11057) = 12.1, *p* <.001). Specifically, Tukey’s HSD comparisons showed that those with the most self-report trust had significantly more negative change in generosity ratings compared with those with the least trust (*p >*.001, mean difference = 4.82, 95% CI [2.50, 7.13]) and in the middle 50% (*p* >.001, mean difference = 3.30, 95% CI [1.19, 3.31]). Upon visual inspection of the plot, this effect seems to be most pronounced in the greatest gain and greatest loss conditions (Fig. 5b). Another ANOVA was conducted to determine whether gender predicted change in generosity rating when controlling for trustee generosity condition, and gender did have a significant effect on generosity rating, *F*(2,11056) = 7.12, *p* < 0.001. Specifically, Tukey’s HSD comparisons showed that male participants had significantly more negative change in generosity ratings as compared to that of female participants (*p* =.0030, mean difference = 1.67, 95% CI [– 2.86, – 0.48]), and that there was also a marginally more negative change in the nonbinary or other participants’ generosity ratings as compared to those of female participants (*p =*.053, mean difference = – 4.05, 95% CI [– 8.14, 0.04]), but no significant difference between nonbinary or other participants’ ratings and male participants’ ratings (*p* =.36). Upon visual inspection of the plot (Fig. 6b), this effect seems to be driven by the variable-gain loss (1.0) and loss (0.5, 0.75) trustee generosity conditions, suggesting that males and nonbinary or other participants rate trustees that behave unfairly some or all the time more negatively than do female participants.

Finally, we examined how investment behavior changed over repeated rounds, whether it stabilized late in the task, and whether participants ceased investing altogether with less generous trustees.

#### Change in investment behavior

The slope of participants’ point investments by trustee generosity condition over the course of the game was calculated to examine change in investment behavior for each type of trustee over rounds (Fig. 4c). Negative slopes indicate decreasing investment across rounds, whereas slopes closer to zero or positive indicate stable or increasing investment over time. Accordingly, we expected more negative slopes for low-generosity trustees and less negative or positive slopes for high-generosity trustees. To balance interpretability with appropriate handling of the repeated-measures structure of the data, learning slopes were first estimated as descriptive summaries of investment change over time and were then analyzed using linear mixed-effects models to test group-level differences while accounting for within-participant clustering.

Mean investment was calculated for each combination of round number, trustee generosity condition (0.5, 0.75, 1.0, 1.25, 1.5), and gender. Standard errors of the mean (SE) were computed using the standard deviation of investment divided by the square root of the number of unique participants in each grouping. To investigate investment behavior progression across rounds, rounds were categorized into the four blocks (1–10, 11–20, 21–30, 31–40). Linear regressions were conducted within each of these round ranges to predict mean investment as a function of round range for each combination of trustee generosity and gender. The slope of investment change over rounds was extracted from each model, along with its standard error. To account for variability across groups, a pooled standard error of the slope was calculated by averaging the within-group standard errors, ensuring a more stable estimate of investment trends across conditions.

To determine whether the effect of trustee generosity on investment slopes differed by gender, a linear mixed-effects model was conducted to examine the effects of trustee generosity on the slope of participant investment across rounds, accounting for random intercepts for each participant. The model revealed the expected significant main effect of trustee generosity condition, indicating that as trustee generosity increased, the slope of participant investment became less negative (i.e., participants were more likely to maintain or increase their investment over time). A significant main effect of gender was also observed, with male participants exhibiting significantly more negative investment slopes than female participants, *B* = − 0.102, *SE* = 0.019, *t*(1,700) = − 5.38,* p* <.001. The nonbinary or other gender category did not significantly differ from females, *B* = 0.064, *SE* = 0.065, *t*(1,700) = 0.98, *p* =.327. A significant interaction between trustee generosity condition and gender was found for some conditions, suggesting that the effect of trustee generosity condition on slope differed by gender (Fig. 6c). Specifically, in the 1.25 condition, males showed significantly higher investment slopes compared to females, *B* = 0.073, *SE* = 0.020, *t*(10,500) = 3.67, *p* <.001. This suggests that while males initially showed steeper declines in investment, they became more stable when interacting with more generous trustees. In the 1.5 gain condition, males again exhibited significantly higher slopes than females, *B* = 0.054, *SE* = 0.020, *t*(10,500) = 2.70, *p* =.007. For the 0.75 and 1.0 conditions, male participants’ investment slopes were not significantly different from those of females (*p* >.05), indicating that the gender differences mainly emerged at the highest generosity levels. None of the interactions were statistically significant for the nonbinary or other participants (*p* >.05), indicating that nonbinary or other participants did not significantly differ in how their investment slopes changed across different trustee generosity levels. Overall, male participants had steeper declines in investment over time compared to females, except at the highest generosity levels (1.25 and 1.50), where their slopes became significantly more stable. Additionally, increasing trustee generosity was associated with higher investment slopes for all participants, suggesting that gain-condition trustees encouraged participants to maintain or increase their investments over time.

The same procedure was followed to examine the effects of self-reported trust, trustee generosity condition, and their interaction on investment slopes over time (Fig. 5c). Self-reported trust had a significant negative effect on the investment slope, *B* = – 0.013, *SE* = 0.005, *t*(1635) = 2.71, *p* =.007, suggesting that high self-reported trust is associated with slightly lower declines in investment over time, which indicates that more self-reported trust may reduce the tendency to decrease investment in this task. The interaction between self-report trust and trustee generosity condition was significant in the variable gain-loss (1.0: *B* = 0.020, *SE* = 0.005, *t*(10500) = 3.99, *p* <.001) and gain conditions (1.25: *B* = 0.020, *SE* = 0.005, *t*(10500) = 2.08, *p =*.038; 1.5: *B* = 0.020, *SE* = 0.005, *t*(10500) = 1.91, *p =*.056). These results suggest that participants with more self-report trust increase their investments more when interacting with more generous trustees.

#### Investment plateaus

The average point value of participants’ last three investments was calculated for each trustee generosity condition. A series of ANOVAs was performed to examine whether self-report trust quartile significantly predicted participants’ investment plateaus for each trustee generosity condition. While there were no significant differences between average plateaus in the loss conditions (0.5: *F*(2,550) = 1.41, *p* = 0.246; 0.75: *F*(2,550) = 2.86, *p* = 0.058) by self-report trust quartile, in the variable-gain loss condition, there was a significant difference in average plateau values by self-report trust quartile, *F*(2,550) = 10.36, *p* < 0.001. Specifically, those with the least self-report trust had significantly lower plateaus than those in the middle 50% (*p* <.01), and significantly lower plateaus than those with the most self-reported trust (*p* <.001). Those with the least self-report trust also had significantly lower plateaus (*p* =.041) than those with the most self-report trust in the 1.25 gain condition (*F*(2,550) = 3.34, *p* = 0.036), but no other comparisons were significant in the gain conditions. Again using ANOVAs, gender did significantly predict participants’ investment plateaus in gain conditions (1.25: *F*(2, 550) = 5.906, *p* = 0.0029); 1.5: *F*(2, 550) = 5.982, *p* = 0.0027), as well as in the 0.75 loss condition (*F*(2, 550) = 3.764, *p* = 0.024); however, in the 0.5 loss condition (*F*(2, 550) = 2.977, *p* = 0.052) and the variable gain-loss condition (1.0: *F*(2, 550) = 0.527,* p* = 0.59), there were no significant differences in investment plateau by gender. In the gain conditions, male participants had significantly higher plateaus than female participants (1.25: difference = 0.66, *p* = 0.0021; 1.5: difference = 0.56, *p* = 0.002), whereas in the significant loss condition male participants had significantly lower plateaus than female participants (0.75: – 0.48, *p* = 0.036) while non-binary and other participants did not show significant statistical differences in investment plateaus across trustee generosity conditions compared to male and female participants.

#### Desisting from investing

To probe if and when participants ceased to invest at all by trustee generosity condition, especially in cases of unfair trustees, the number of zero investments—in which participants did not invest any points at all with a trustee—per round was calculated for each generosity condition. To be able to compare subgroups with uneven numbers of participants, the percentage of participants *still investing* per trial was calculated as number of non-zero investments divided by the total number of participant investments on each trial (Fig. 4d). Percentages were calculated for each trustee generosity condition and round number, and averaged across blocks (e.g., the final investment percentage for Round 1 was averaged from the investment percentage of the first decision of the first block, second block, third block, and fourth block). These plots were then subdivided by self-reported trust and gender. Upon visual inspection of the self-report trust plot, the percentage of the most trusting individuals that continued to invest in the task in the loss and variable gain-loss conditions was higher than the percentage of lower trust participants that continued to do so (Fig. 5d). Additionally, the percentage of female and nonbinary or other participants that continued to invest in the task in the loss and variable gain-loss conditions appeared higher than the percentage of male participants that continued to do so (Fig. 6d).

### Participant feedback

#### Trustee believability

After completing the task, participants were asked to respond to “While playing the game, did you believe that you were genuinely playing with past research participants, or something else?” using a slider bar from “I definitely did not believe I was playing with past research participants – I believed something else” (coded as – 10) to “I definitely believed I was playing with past research participants” (coded as 10). All 553 participants responded to this item, and their composite responses corresponded with overall slight disbelief but high variability (*M* = – 1.10, *SD* = 6.35). Participants were then given the option to respond in a free-response box to “If you believed something else: what did you believe?” Free responses were thematically coded, and counts of each response category were produced (Table 1).

**Table 1:** Responses to “If you believed something else: What did you believe?”

Response Category	Response Counts	Percentage of Sample
The participant did not indicate that they did not believe the task instructions	346	61.6%
The task was ‘Organized’ or ‘controlled’ in some way	2	0.4%
‘Preprogrammed’ or ‘programmed’ or ‘scripted’ in some way/I was playing with a computer	88	15.7%
AI/artificial intelligence/bots	59	10.5%
The researchers/the task was playing with the researchers	27	4.8%
I was uncertain (broadly defined)	24	4.3%
The task was algorithmic (eg. Trustee responses were based on my responses) or algorithmic based on responses from past participants; some as specific as ‘each character was assigned a return % with a small range”	33	5.9%
Randomized responses	26	4.6%
‘Fictional characters’/‘not real people’/avatars	20	3.6%
Unknown/indecipherable based on response phrasing	11	2.0%
I was set up to lose	1	0.2%
I believed the responses were generated based on past participants but that the responses were matched with a random face, or I'm unsure if these past responses were made by the people in the photos	6	1.1%
The response addressed something about the photos, including: photos were or looked “AI generated”; photos were of random people; photos were too high quality to have been photos of previous participants	40	7.1%
I believed the other players were confederates (defined as people in confederation with the researchers playing the role of the other participants in the game)	1	0.2%
The response addressed something about race/gender biases or prejudices	11	2.0%
Deception: any other response	8	1.4%
I believed I was playing with new participants (not previous participants)	1	0.2%

Of the 553 participants, 246 wrote in the free response box, and 14 participants wrote either “N/A” or the equivalent. Free responses were thematically coded, and counts of each response category were produced. *Note:* participant responses could contain themes from more than one category, which is represented in these counts

#### Trustee distinctiveness

Participants were asked “When you played this game, were there any faces that you were more drawn to than others? Any that were memorable for a specific reason?” Free responses were thematically coded, and counts of each response category were produced. Of the 553 participants, 230 were not drawn to specific faces or did not find any more memorable than others (Table 2).

**Table 2:** Thematically coded participant responses to “When you played the game, were there any faces you were more drawn to than others? Any that were memorable for a specific reason?”

Response Category	Response Counts	Percentage of Sample
No, or not particularly	233	41.5%
Yes (unspecified), or yes (a certain one of the people, unsure of the reason or reason unspecified)	29	5.2%
Those with big eyes felt they would be more generous (or were more memorable); alternately, those whose eyes were more open	12	2.1%
I paid more attention to the faces that were more generous during the game/generous faces were more memorable	43	7.7%
I trusted kind/genuine/warm/friendly faces more/kind eyes, or I was drawn to kinder/genuine faces but that didn’t influence how generous they were	55	9.8%
I trusted happy/more positive faces more/was drawn to happier faces/smiles	35	6.2%
I trusted attractive faces more/thought attractive faces were more generous/trusted those with more symmetrical features more	18	3.2%
People resembled real life friends/faces that seemed familiar; also, trustees in the game that looked like earlier trustees that had been generous	14	2.5%
Distinguishing feature/features: Some faces were misshapen and easier to remember/their face or neck shape, or a feature they had (e.g., specific eyebrows) made them memorable/facial features impacted how much I trusted someone or how much I was drawn to them	33	5.9%
I trusted frowning faces less	5	0.9%
I'm unsure	2	0.4%
I thought women would be more generous/was drawn to more feminine faces	25	4.4%
I didn’t like the ugly ones	1	0.2%
The ones that looked closer to me	1	0.2%
Participant did not answer the question (commented on a different part of the task) or their response was unclear	26	4.6%
I was more drawn to someone similar to me (e.g. Asian faces because I’m Asian, women because I’m a woman, my own race)	15	2.7%
I found more assertive faces less trustworthy because they were likely focused on maximizing their profit	1	0.2%
Some faces looked angry/hostile/aggressive/intentionally rude/annoyed/stern/hard/cold/in a bad mood/unhappy/stone-faced; (and in some cases) I trusted angry/aggressive people less	25	4.4%
I was drawn to people with softer, rounder features/drawn to certain face shape	21	3.7%
Wider faces seemed more genuine	1	0.2%
Facial expressions influenced me, e.g., people with a natural expression seemed to look more trusting (could have meant ‘trustworthy’); people with a softer facial expression looked more trustworthy	20	3.6%
I remembered the faces that could **not** be trusted more	12	2.1%
I was more drawn to the Asian characters; e.g., and believed they were more trustworthy, and they were. The white characters were the worst performers, and the black characters gave neither good nor bad points; e.g., I was more drawn to the asian and black faces, and I found that while playing the game the white people seemed to be less generous; e.g., drawn to the ‘asian women’ in the fourth trial	13	2.3%
I was drawn to more unique faces	1	0.2%
Some [faces? people?] felt more closed, and therefore less generous	2	0.4%
The ones that showed stronger emotions were the most memorable/the ones with more self-confidence	2	0.4%
I remembered faces that e.g. looked stern but were actually generous	3	0.5%
Confirmation bias: people were memorable if I thought they looked like they were going to be generous, and then they were or the other way around	4	0.7%
I thought the men were more memorable	1	0.2%
I thought the younger a person was the more generous they would be	1	0.2%
’Stronger’ features elicited an emotion of caution	1	0.2%
I was most drawn to the people I rated as most generous during the rating part of the task	1	0.2%
POC profiles were more memorable to me	1	0.2%
I was drawn specifically to the black women	1	0.2%

Participant responses could contain themes from more than one category, which is represented in these counts

#### Participant engagement

**Fig. 7:**
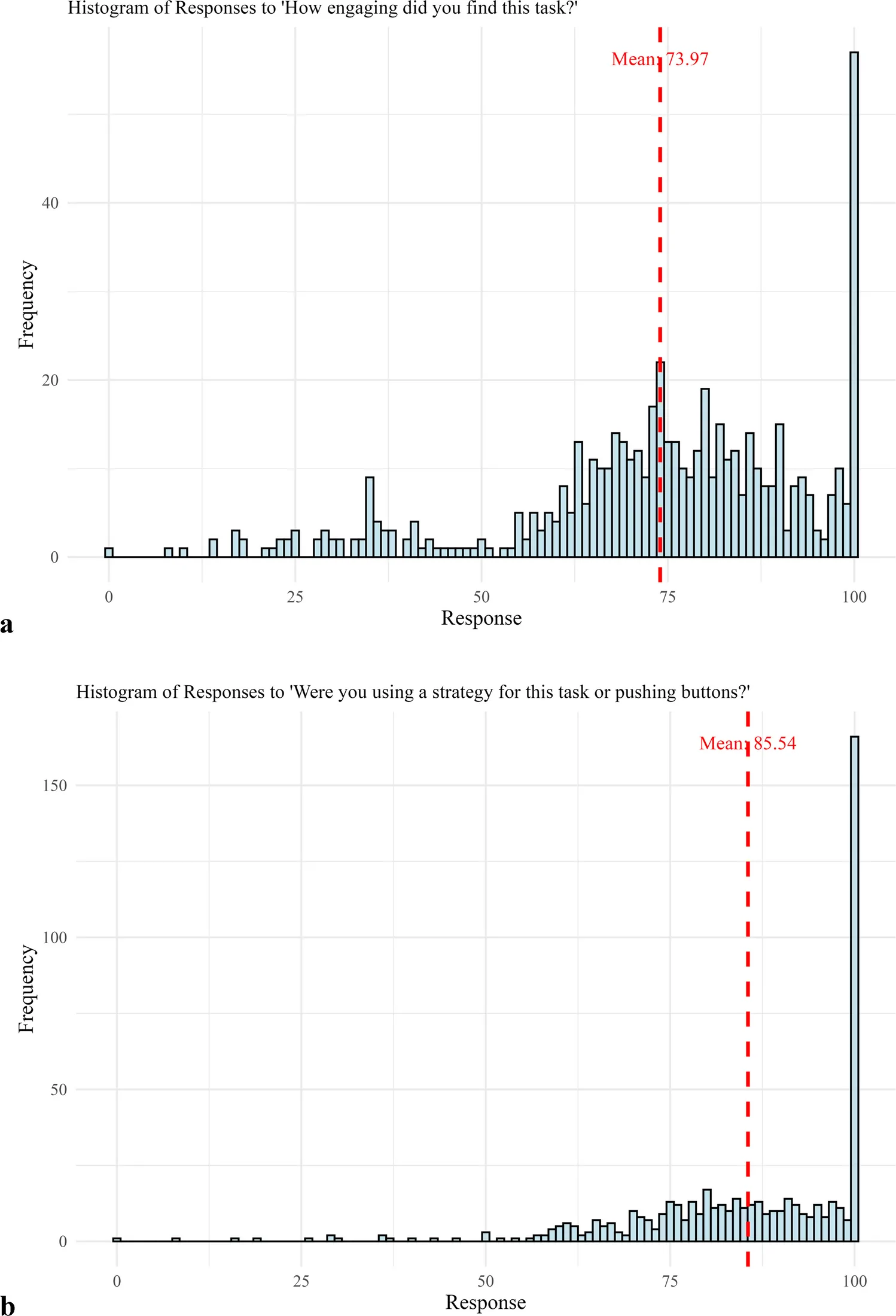
**a** Histogram of participant responses to “How engaging did you find the task?”; **b** Histogram of participant responses to “Were you mostly using a strategy for this task, or mostly just pushing buttons to try to get the task to end as quickly as possible? (Don’t worry, your answer won’t affect your study payment; we just want to learn about how people are interacting with this task!)”

Participants responded to “How engaging did you find this task?” using a Likert scale from “extremely unengaging” (coded as 0) to “extremely engaging” (coded as 100; *M =* 74.0, *SD* = 20.1). Participants next used a slider bar to answer “Were you mostly using a strategy for this task, or mostly just pushing buttons to try to get the task to end as quickly as possible? (Don’t worry, your answer won’t affect your study payment, we just want to learn about how people are interacting with this task!)”. Responses were recorded from “Just pushing buttons to get the task to end as quickly as possible” (coded as 0) to “Genuinely using a strategy to try to win as many points as possible” (coded as 100; *M =* 85.5, *SD* = 15.8), and indicated high task engagement overall. For histograms of participant responses, see Fig. 7.

#### Research purpose

Participants had the option of responding to “What do you think we were studying in this research?” 340 of the 553 participants chose to respond to this question. Free responses were thematically coded, and counts of each response category were produced (Table 3). The most frequent response types suggested the belief that this research investigated something about race or ethnicity, gender, or a combination of both, or that this was a study of discrimination or stereotyping based on people’s appearances.

**Table 3:** Thematically coded participant responses to “What did you think we were studying in this research?”

Response Category	Response Counts	Percentage of Sample
Not sure, no idea, or N/A	21	3.7%
Something about **race**/ethnicity/racial prejudices	125	22.2%
Something about **gender**	42	7.5%
If race and/or gender impacted generosity ratings/trust	88	15.7%
**Social investment/** how do [young] people trust strangers when it comes to monetary/profit decisions; discerning the trustworthiness of strangers	22	3.9%
Seeing if people who are more **attractive** are given more chances after being greedy/impact of attractiveness on judgement of generosity; how beauty affects how much you trust someone	3	0.5%
The give and take nature of human relationships/**interpersonal trust/trust, unspecified**/how we relate to others/interpersonal judgement unspecified	34	6.0%
People’s discrimination/trust/perceptions/stereotypes in light of people’s **appearance** (broadly/not specified further, or “certain features”, “certain characteristics”, “different demographics”, “faces”)	111	19.8%
**Biases**, broadly defined; looking at people’s perceptions initially based on appearance versus their actual generosity	29	5.2%
To see if **mood** is impacted when they realize they judged someone correctly/incorrectly; level of emotion from before to after doing a decision-making task; **mood** as impacted by the task	7	1.2%
To see if trust is affected when participants are given a task to earn the most points	1	0.2%
**Indecipherable respons**e or response to a different question	9	1.6%
How people change their investment decisions based on past results/how they are treated;evolution of strategy; learning responses	29	5.2%
The relationship between mental health and decision-making/trust; how one impacts the other and vice versa	12	2.1%
Explicitly that we attempt to influence participants’ opinions by ensuring that some trustees the participant rated as more likely to be generous were ungenerous, thereby attempting to eliminate some of the stereotypes the participant had in mind/more broadly, **how perceived perception and actual result changes a person’s way of thinking**	8	1.4%
To see if trust is influenced by people’s **facial expressions**	6	1.1%
To see how **first impressions** influence perceptions of generosity	17	3.0%
Perceptions and how people judge other people, broadly defined	6	1.1%
What demographic I relate to most	1	0.2%
The psychology of kindness	1	0.2%
**Investigating decision-making** and its association with specific dispositional constructs; investigating **decision-making** and **risk-taking**	11	2.0%
Studying how many times one is willing to invest in someone before deeming them untrustworthy/how do investors respond when they feel like their trust has been betrayed	5	0.9%
To see how many points we could get	1	0.2%
How people interact and** make decisions when it involves affecting another person**	1	0.2%
Trust in **humanlike AI systems**	1	0.2%
If people trust **people who look like them** more/correlation between how we judge others based on their appearance and how that correlates to participant’s own demographic	3	0.5%
Game theory	1	0.2%
Displaying how the government proportions money based on return as opposed to need.	1	0.2%

Participant responses could contain themes from more than one category, which is represented in these counts; 340 participants elected to respond to this question

## Discussion

The SL-Gen task complements and extends existing multi-round trust game paradigms (e.g., Chang et al., 2010; Van Den Bos et al., 2011; Vermue et al., 2018, 2019; Ewing et al., 2015) by offering a standardized, scalable, and potentially more ecologically valid framework for examining feedback-driven social learning and generosity sensitivity. Unlike previous designs that focused on facial trustworthiness cues, binary trust decisions, or symbolic partners, the SL-Gen employs realistic, demographically diverse facial stimuli, randomized trustee–condition assignments, and a continuous investment scale, enabling a finer-grained and more lifelike characterization of social decision-making.

This task was developed to characterize social exchange behavior among young people in a nuanced, ecologically realistic manner. This population-level step is critical for establishing a baseline for comparison when examining social learning and decision-making in young people across healthy and clinical populations. Specifically, a better understanding social learning, generosity sensitivity, and trust-based decision-making in young people can help elucidate mechanisms of social function or dysfunction in situations and populations where social functioning is disrupted. Taken together, the task outcomes provide complementary indices of social learning and generosity sensitivity, with some measures being more proximal to one process than the other.

Simultaneously, it was important to demonstrate that this task shares features with existing versions of the trust game, namely, that it is sensitive to gender and self-reported trust effects previously observed in research with the trust game. While the SL-Gen task retains the interpersonal structure of the multi-round trust game, it focuses on feedback-driven social learning rather than strategic reciprocity, using pre-programmed trustee “generosity” parameters to capture how individuals adapt to consistently fair or unfair partners over repeated interactions.

Indeed, recent meta-analytic work by Duncan and colleagues (2025) on repeated trust-game paradigms provides an important broader context for the present findings. Across studies, the authors report that the strongest driver of trust learning is partners’ behavioral trustworthiness, with reciprocation rate emerging as the clearest predictor of how much participants adjust their investment behavior over time. This pattern aligns closely with the SL-Gen task, where participants differentiated strongly across generosity conditions and updated both their investments and generosity ratings over repeated interactions. By contrast, other features such as demographics and several task design variables show smaller or more inconsistent effects (Duncan et al., 2025). In that sense, the SL-Gen paradigm appears to preserve the core learning phenomenon identified in the broader repeated-trust-game literature, while offering a more structured way to examine how that learning unfolds across distinct social-exchange conditions. The repeated trust-game literature reviewed also helps clarify why it is useful to distinguish between early investment tendencies and later learning dynamics. This recent review suggests that many studies capture either initial trust or average investment, but not the change in behavior over time that constitutes trust learning itself. The design of the SL-Gen task addresses the nature of that gap by separating initial investments, investment slopes, plateaus, and disengagement, which makes it possible to ask not only whether participants trust more or less overall, but how they update behavior as they receive more information about others’ generosity across rounds.

### Gender

In this study, being female was associated with investing fewer points both initially and overall and earning fewer points in the SL-Gen task. With respect to trustee generosity conditions, female participants invested fewer points in the gain conditions (1.25 and 1.5) and the variable gain-loss condition (1.0) than males, and exhibited less of a negative change in generosity rating in loss conditions (0.5 and 0.75) and the variable gain-loss condition (1.0) than did males. Being female was also associated with a blunted negative change in investment pattern in loss conditions as compared to the investment pattern of males, and female participants continued to invest for longer in variable gain-loss and loss conditions than male participants. Taken together, these gender effects were evident across outcomes that most directly index both social learning and generosity sensitivity. These results mirror and add nuance to the gender effects reported in the van den Akker and colleagues (2020) meta-analysis, both in terms of point investment patterns and social risk. Additionally, no previous research to our knowledge, has included an analysis of nonbinary or other gender identity decision-making in a social exchange task context; while this study had a small sample size of nonbinary or other participants (*n* = 12), these data are reported for transparency and inclusivity and should be considered exploratory. The overall pattern of results was unchanged when analyses were restricted to male and female participants, suggesting that inclusion of the nonbinary group did not meaningfully influence the main findings. Nevertheless, these data provide a useful initial reference point for future studies, including this population.

### Self-report trust

The SL-Gen task also extends and adds clarity to the relationship between self-reported trust and trust game behavior (Banerjee et al., 2021). Specifically, higher self-reported trust was associated with higher initial investments and more points invested in all trustee generosity conditions in this task. Participants with higher self-reported trust showed a significantly more negative change in their generosity ratings than those with lower self-reported trust, especially in the greatest gain and loss conditions, suggesting that those who trust others more have stronger reactions to others’ perceived lack of generosity. Higher self-reported trust was associated with smaller declines in investment behavior over time, and participants with greater self-reported trust increased their investments more when interacting with more generous trustees. Those with higher self-reported trust had higher investment plateaus in the variable gain-loss and 1.25 gain conditions, and a greater percentage of high self-reported trust participants continued to invest in the task in the loss and variable gain-loss conditions than did low self-reported trust participants. Taken together, more self-reported trust was associated with greater investment for longer and with greater sensitivity to being treated unfairly in a social exchange task setting, consistent with behavior relevant to both social learning and generosity sensitivity.

### Task features and acceptability

While this task is modeled on existing versions of the trust game, it is novel, and it was important to obtain participant feedback on its features. In general, participants seemed to find this task enjoyable and engagement in completing the task was high. Despite efforts to phrase the question of whether participants believed they were playing with past participants or something else as neutrally as possible, the phrasing of the question (or indeed, its existence) may have skewed respondents toward thinking that they were not playing with past participants, so it is possible that those reporting that they thought something else are overrepresented. Future use of this task should consider phrasing this question more neutrally.

#### Trustee facial expressions

While we selected neutral faces for this task, some participants identified the trustees as looking angry, hostile, aggressive, intentionally rude, annoyed, stern, hard, and cold. This could be a negative emotional processing bias (as other participants explicitly reported that the images “all had neutral expressions” or “resting neutral faces”), but future research with this task or similar tasks could consider using images with slight smiles.

#### Artificial intelligence

Data collection for this study was conducted close to the time of the public release of ChatGPT, and quite a few participants reported that they believed this study was investigating something about interactive artificial intelligence, or that they were playing the game with AI. While this does not directly impact the study outcomes reported here, it is perhaps useful for researchers conducting online psychological studies via platforms like Prolific to be aware that individuals may believe social interaction tasks have an AI component.

#### Race, gender, and bias

Many participants reported believing that we were studying something about race, gender, or bias. In this task, we race- and gender-balanced trustees evenly, and trustee faces were randomly assigned to generosity conditions each time the task was generated. This design ensured that trustee race and gender were not systematically associated with any behavioral condition, helping to minimize potential bias in the task.

That said, the possibility remains that participants’ perceptions or investment decisions could have been influenced by the ethnicity of the trustees or by participants’ own demographic characteristics. Our participant sample was broadly representative of the UK population aged 18–24 but not demographically balanced across ethnic groups (White = 371, Black = 41, Asian = 97, Mixed/Other/Prefer not to say = 44), which limits the feasibility of formally examining ingroup or outgroup effects. Future work using the SL-Gen task could explicitly investigate how participant and trustee ethnicity interact to shape social learning and investment behavior, ideally using more balanced samples and with the goal of better understanding potential ingroup biases in cooperative decision-making.

More broadly, the fact that many participants commented on the multi-racial and gender elements of this task—often assuming that these features must be central to the research question—suggests that there is a lack of diverse stimuli being used in psychological tasks of this nature, and highlights an ongoing gap in the field. Effort should continue to be made to have multiple ethnicities and genders represented in psychological task stimuli for greater ecological validity and to ensure that participants’ social inferences are grounded in realistic, representative social contexts.

#### Facial recognition

In self-report responses, some participants acknowledged that they were much better at recognizing names and worse at recognizing faces; our task does not account for prosopagnosia, facial aphasia, or individuals that have a difficult time recognizing faces. Future research of this nature could include an item or scale to account for differences in the ability to recognize faces.

### Future directions

The SL-Gen task is a rich measure that offers opportunities to explore future analyses in a multitude of ways and across healthy and clinical populations. For instance, while the present study does not include measures of risk-taking, risk aversion, or altruism—and these and other mechanisms are likely to influence investment behavior—future research with the SL-Gen task could seek to disentangle these contributing mechanisms more precisely.

Additionally, to more directly examine social learning and generosity sensitivity and their relationship, future research could include longitudinal paradigms to investigate these two constructs more separately, or investigate across multiple time points whether differences in generosity sensitivity are associated with differences in how quickly social learning unfolds, or vice versa. Computational modeling approaches may also be especially useful in separating learning rate, choice consistency, sensitivity to unfairness, and other contributors such as risk or inequity aversion, or examining social learning and generosity sensitivity as interacting but partially dissociable components of social exchange, rather than only as complementary behavioral indices within a single cross-sectional task. Future work could specifically build upon the finding of Duncan and colleagues’ (2025) review by testing whether individual differences in generosity sensitivity shape the speed or stability of learning across repeated social interactions, and whether these processes remain stable across longitudinal or treatment-related reassessments.

In light of existing research suggesting that social exchange and cooperation-based task behavior can be influenced by treatment (Marsh, 2021; Bibbey, 2020), both via psychological interventions (Williams et al., 2014) and medication, such as SSRIs (Levy et al., 2014), future research could include this task to probe for these differences in mental health treatment contexts. However, as the present study used a single-session design, the SL-Gen task’s test–retest reliability, longitudinal stability, or susceptibility to practice effects have not been established. This is particularly important if the task is to be used in future clinical or treatment studies as a repeated pre–post outcome measure. Although the task structure may reduce within-session carryover by presenting new trustee faces in each block and randomizing faces to generosity conditions, this does not replace formal test–retest evaluation. Future studies should examine the stability of SL-Gen outcomes across repeated administrations, quantify practice effects over clinically relevant intervals, and determine which task indices are most stable versus most sensitive to treatment-related change. Although formal test-retest and practice-effect studies are still needed, the present findings suggest that the SL-Gen task is a promising candidate paradigm for future work seeking to characterize how social learning and generosity sensitivity may differ across individuals, life stages, and clinical contexts.

## Supplementary Information

Below is the link to the electronic supplementary material.

Supplementary file1 (DOCX 3229 KB)

## Data Availability

All data can be found online here: osf.io/2t7md/; the *Social Learning-Generosity (SL-Gen) Task* is available on Gorilla’s Open Materials at: https://app.gorilla.sc/openmaterials/1045631. This project was not preregistered.

## Footnotes

1 Of these, seven participants wrote “non-binary”; one wrote “non-binary trans masc”; two wrote “genderqueer”; one wrote “transgender male”; one wrote “non-descript”; and one wrote “non-conforming”.

## Code availability

The code from the statistical analyses for this paper is available here: osf.io/2t7md/.
