# Supplementary material for: The Social Learning-Generosity (SL-Gen) Task: Redesigning the multi-round trust game paradigm to examine social learning and generosity sensitivity in young people
Source: Behav Res Methods. 2026 Jul 24;58(9):249. doi: 10.3758/s13428-026-03100-2 (PMC13400464; doi:10.3758/s13428-026-03100-2)
Supplement: Supplementary file 1 — Supplementary file1 (DOCX 3229 KB) [file 13428_2026_3100_MOESM1_ESM.docx]

**Supplementary Materials for**

**The Social Learning-Generosity (SL-Gen) Task: Redesigning the multi-round trust game paradigm to examine social learning and generosity sensitivity in young people**

***Table S1.* A Selection of Existing Trust Game Paradigms, Uses, and Examples.**

******

***Table S2*. Expanded demographic information for participants.**

***Pre-Game Trustworthiness Ratings of Trustee Faces***

Participants were asked to rate on a scale from 1-100 how generous they thought each trustee would be before completing the ten rounds of the SL-Gen task with that trustee. As faces convey a lot of information that individuals can use to make decisions in the trust game (Chang et al., 2010; Todorov et al., 2009; Willis & Todorov, 2006), the pre-game generosity ratings for each trustee face were computed as the average pre-game generosity rating across all 553 participants per face.


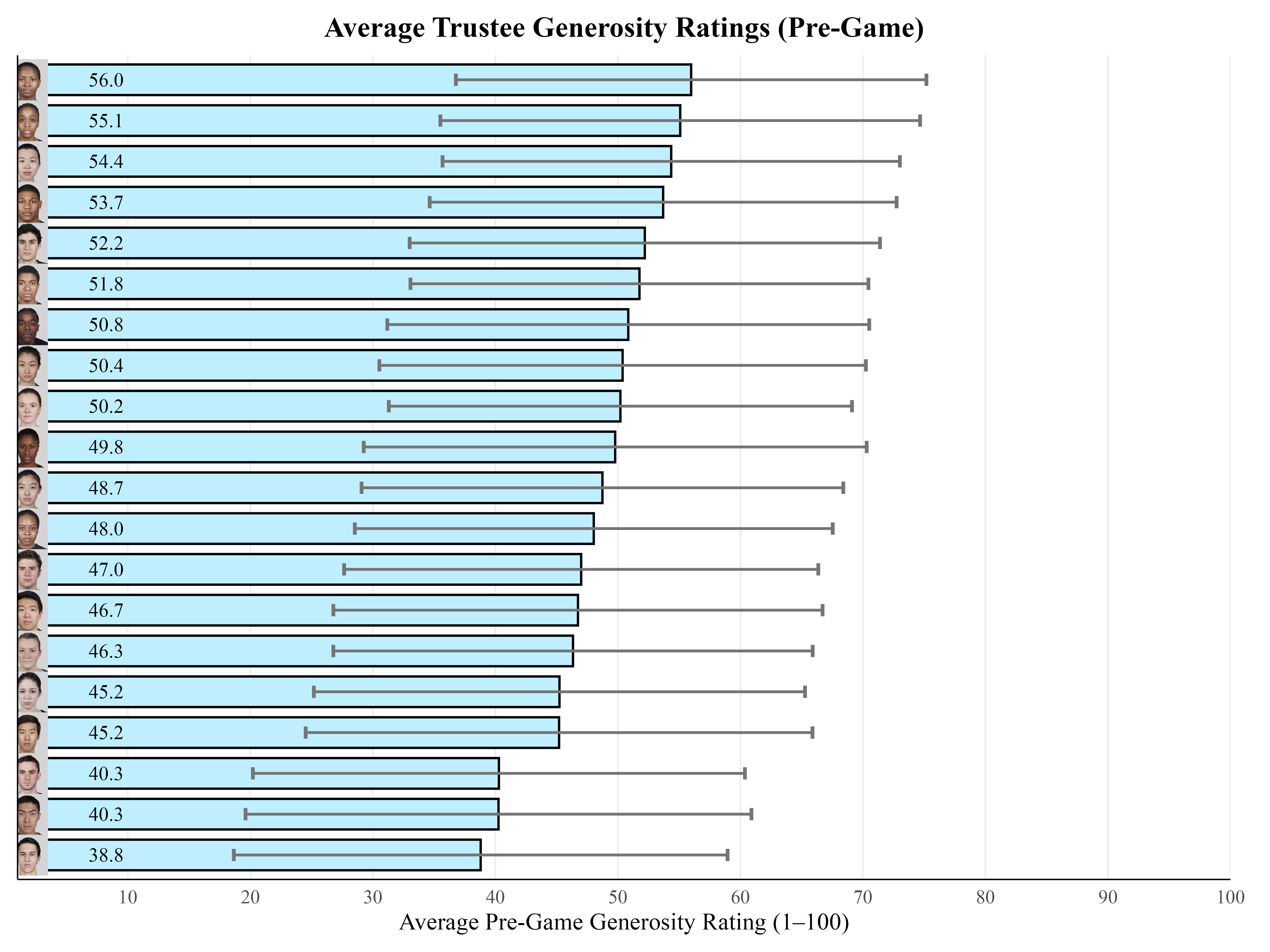


***Figure S1.* Average pre-game generosity ratings of all trustees for all 553 participants with standard deviation bars.**


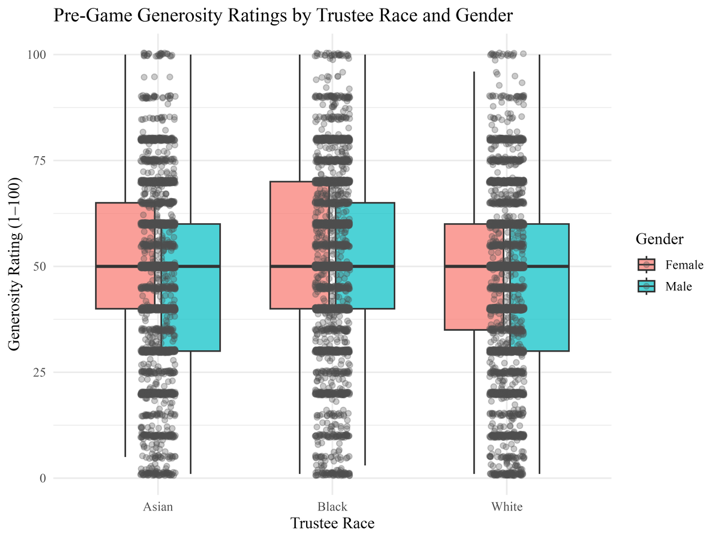


***Figure S2.* Pre-game generosity ratings by trustee race and gender.**

A linear mixed-effects model with participant as a random intercept revealed significant differences in pre-game generosity ratings by trustee race and gender. Relative to Asian female trustees (the reference group), Black female trustees were rated slightly more generously, β = 1.06, SE = 0.50, *t*(10 502) = 2.11, *p* = .035, whereas White female trustees were rated significantly less generously, β = –3.90, SE = 0.54, *t*(10 502) = –7.26, *p* < .001. Male trustees were rated less generously overall, β = –7.09, SE = 0.54, *t*(10 502) = –13.21, *p* < .001. The significant Race × Gender interactions indicated that the gender effect differed by race: for Black trustees, the male–female difference was smaller (β = 6.97, SE = 0.74, *t*(10 502) = 9.48, *p* < .001), and for White trustees, males were also rated relatively more generously than females (β = 4.41, SE = 0.74, *t*(10 502) = 5.99, *p* < .001). There was substantial between-participant variability in overall generosity ratings (SD = 12.6) and moderate within-participant residual variability (SD = 15.5).

After the game, these racial and gender differences were reduced. In the post-game model, there were still significant main effects of trustee race and gender but with attenuated magnitudes: White female trustees remained rated less generously than Asian female trustees, β = –3.54, SE = 1.09, *t*(10 504) = –3.26, *p* = .001, and male trustees were still rated lower overall, β = –2.67, SE = 1.09, *t*(10 504) = –2.45, *p* = .014. However, there was no significant difference between Black and Asian female trustees, β = 0.07, SE = 1.02, *t*(10 504) = 0.07, *p* = .95. Race × Gender interactions remained significant, such that for both Black trustees (β = 3.81, SE = 1.49, *t*(10 504) = 2.56, *p* = .010) and White trustees (β = 4.94, SE = 1.49, *t*(10 504) = 3.32, *p* = .001), the gender difference was smaller or reversed post-game. Random-effect estimates indicated continued between-participant variability (SD = 8.39) and increased residual variance (SD = 31.34).

Together, these results suggest that participants initially made stronger demographic-based distinctions in perceived generosity, but that these differences diminished after gameplay, indicating that participants judged individual trustees more on their observed behavior than on demographic cues.

***Summary of statistical analyses and rationales***

**Note**: Analyses were selected according to the level of each outcome variable and the validation question being addressed. The aim was to characterize interpretable task behavior across distinct phases of the SL-Gen task rather than to fit a single comprehensive model to all trial-level data. Participant-level summary outcomes were analyzed using participant-level ANOVA or regression models. Outcomes summarized by trustee generosity condition were analyzed either condition-wise or with trustee generosity included as a categorical task condition, reflecting the task design in which generosity levels represented distinct loss, variable gain/loss, and gain contexts. Mixed-effects models were used where repeated observations were central to the research question, particularly for investment-slope analyses. Desisting from investing was treated as an intentionally descriptive visualization analysis rather than a formal inferential test. Because the study was exploratory and validation-focused rather than preregistered, results are interpreted based on the coherence of patterns across complementary outcomes rather than family-wise corrected significance testing.

***Table S3.* Analysis method and rationale for each analysis (continued on the next page)**

***Table S3 (continued).* Analysis method and rationale for each analysis.**
